# Supplementary material for: Chromothripsis during telomere crisis is independent of NHEJ, and consistent with a replicative origin
Source: Genome Res. 2019 May;29(5):737–49. doi: 10.1101/gr.240705.118 (PMC6499312; doi:10.1101/gr.240705.118)
Supplement: Supplemental Material [file supp_gr.240705.118_Supplemental_file_1.zip › contigs/annotated_contigs/DB56/contig.2.DB56_length_485_mean_cov_5.7381443299.docx]

**DB56_length_485_mean_cov_5.7381443299**

```
TCATGATATCATCTGAATCTAATTTCCTTCCAAAGGTCCCACCACGTATTAGCATCCCGTTGGATGCTATCACCTGAGCCACAGGTGAA
 >chr7:147059996-147060251 - E=6e-142 p=0e+00
GGTTCAGACCCACTGACCCATTGGAGGTGAGGGTTTCATCTTGATTGTACCTCAAATTCATGGATCAGTATAAATCTGATTCAGTCATC

ACTGAATAGACTCTTTGATAAAGCATATATTGTTATCAACTCTGTCTTGAAGTCATTTCCAACTCAATTGACTCTTG|GTCTAGGGAAA

ACTCAATTGACTCT|TGGTCTAGGGAAAACTGGTTCGAACATTCCACAACTACTCTTTTCTCTGCTTTTTTGAACTCACTGTACACTTT
               >chr7:144278467-144278616 - E=5e-78
CAAAAATATTTTTAAGTAATTCTTGTCTTTTAAAACCCTACTCAAAATTTTTGTCTTCTATGGAGCATTTCCAAA|AAATCGGAATAGA

GACGTGTAGTGACAGGGTGTGTTACTTTCTGTAGGTGTCGTTG
```
